# Impacts of Visualizations on Decoy Effects

**DOI:** 10.3390/ijerph182312674

**Published:** 2021-12-01

**Authors:** Yuin Jeong, Sangheon Oh, Younah Kang, Sung-Hee Kim

**Affiliations:** 1 Management of Technology, Yonsei University, 50 Yonsei-ro, Seodaemun-gu, Seoul 03722, Korea; youin.jeong@gmail.com; 2 Department of IT Convergence, Dong-eui University, 176 Eomgwang-ro, Busanjin-gu, Busan 47340, Korea; shoh9179@gmail.com; 3 Underwood International College, Yonsei University, 50 Yonsei-ro, Seodaemun-gu, Seoul 03722, Korea; yakang@yonsei.ac.kr; 4 Department of Industrial ICT Engineering, Dong-eui University, 176 Eomgwang-ro, Busanjin-gu, Busan 47340, Korea

**Keywords:** decoy effect, consumer choice, debias, information visualization

## Abstract

The decoy effect is a well-known, intriguing decision-making bias that is often exploited by marketing practitioners to steer consumers towards a desired purchase outcome. It demonstrates that an inclusion of an alternative in the choice set can alter one’s preference among the other choices. Although this decoy effect has been universally observed in the real world and also studied by many economists and psychologists, little is known about how to mitigate the decoy effect and help consumers make informed decisions. In this study, we conducted two experiments: a quantitative experiment with crowdsourcing and a qualitative interview study—first, the crowdsourcing experiment to see if visual interfaces can help alleviate this cognitive bias. Four types of visualizations, one-sided bar chart, two-sided bar charts, scatterplots, and parallel-coordinate plots, were evaluated with four different types of scenarios. The results demonstrated that the two types of bar charts were effective in decreasing the decoy effect. Second, we conducted a semi-structured interview to gain a deeper understanding of the decision-making strategies while making a choice. We believe that the results have an implication on showing how visualizations can have an impact on the decision-making process in our everyday life.

## 1. Introduction

There is extensive research on showing how decision-making is dependent on the context of the choices given. Inclusion of alternatives in the choice set can alter one’s preference among the others, and such contextual alternatives are called decoys. Decoy effects are prevalent in decision-making situations, such as when purchasing an electronic good, selecting a phone data plan or internet plan. Companies are inherently interested in guiding customers toward higher-margin products and often utilize the decoy effect to maximize their profits. When they price items to create decoys and attract customers to certain options, it is difficult for customers to discern any spurious choices and make an optimal decision. In other words, individual consumers who do not have control over the information could be easily deceived.

**Figure 1:**
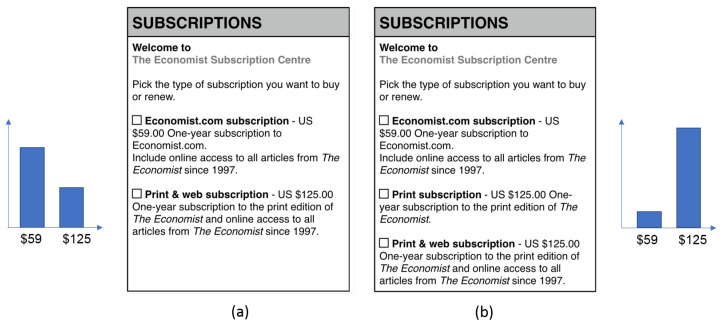
Two subscription pages of the Economist magazine adopted from [1]: (**a**) a subscription page with two common options; (**b**) a subscription page with the two common options and a decoy.

For better understanding, we introduce the well known example below. Suppose that you want to subscribe to the Economist magazine. If you have two options, as shown in Figure 1a, which one would you choose? Then, if you have three options as shown in Figure 1b, which one would you choose? Did you experience differences in your preference regarding options available in the two figures? This is an interesting experiment [1] demonstrating that consumers’ preferences can be manipulated by introducing a seemingly irrelevant option (a decoy; the second option “Print subscription” in Figure 1b). The bar graphs on each side show the percentage of participants who selected a corresponding option. The “Print subscription” option is irrelevant because it is as equally expensive as the “Print & web subscription” ($125) but does not provide online access, and thus is clearly dominated by the “Print and web subscription” option and is called a decoy. By introducing this decoy, the “Print & web subscription” is much more attractive to participants in Figure 1b (84 of 100 votes) than Figure 1a (32 out of 100 votes). This is called the “decoy effect," or ”attraction effect," the phenomenon whereby consumers will tend to have a specific change in preference between two options when presented with a third option that is asymmetrically dominated [2,3,4,5].

Many researchers in behavioral economics, marketing, and psychology have repeatedly studied the decoy effect since it was introduced by Huber et al. [2], which consistently demonstrates flaws in normative decision theories, which claim that decision makers have consistent preferences toward given option [6,7,8]. However, interestingly little research has examined how to break the decoy effect despite the clear benefits for consumers. Therefore, we conducted an experiment to show if visualization can help with debiasing the decision-making process, specifically regarding on the decoy effect. Our research was also partially motivated by Dimara et al.’s study [9,10], wherein the authors found that the attraction effect exists in 2D scatterplots with small number of options; however, the 2D scatterplots could mitigate the attraction effect in a relatively complicated decision-making context (e.g., 10 options). Still, however, is it possible to help with this cognitive bias in a more realistic, conservative decision-making context (e.g., having three options as in Figure 1b)? If we use different types of visualizations, how does it affect the decision-making process?

To address these questions, our first experiment was a quantitative experimental study using a set of visualizations and a real-world dataset to see if visualizations can help alleviate the decoy effect. It was conducted on a crowdsourcing platform as we can recruit a large and diverse group of participants compared to lab experiments [11,12]. Although there are concerns about the data quality, several studies reported that they replicated the results of prior laboratory experiments using crowdsourcing [13,14]. The second study was a qualitative interview study with a smaller set of participants to better understand the decision-making processes while making a choice with a decoy option. We replicated previous findings showing the decoy effect with a tabular representation and tested the effectiveness of a four types of visualizations: one-sided bar chart, two-sided bar chart, scatter plot, and parallel-coordinate plots. After describing the results, we document key findings of the research and discuss several issues. The contributions of our research include:

- It provides empirical evidence that visual interfaces could mitigate cognitive bias in everyday decision-making.
- It provides the first step that different types of visualization could influence the decoy effect differently.
- It provides insights on how different task types and decision-making styles affect the decoy effect.

## 2. Literature Review

### 2.1. Decision-Making and Visualization

Because information visualization techniques can help people comprehend data and transform them into information, several techniques have been used to support the decision-making process. Visual representations and interactions are especially well known ways to help users perceive aspects of data, by augmenting cognitive reasoning with perceptual reasoning and leading to efficient analytic reasoning [15,16].

Data visualization has been utilized for multi-attribute decision-making contexts wherein a decision maker has to select one option from a set of alternatives. The data that need to be considered can easily be organized in a tabular format [17]. Because comparing attribute values is important, parallel-coordinate plots have been used because they can easily project high-dimensional data into a two-dimensional space [18]. The representation is effective because it provides an overview and helps compare values. However, parallel-coordinate plots have a shortcoming lacking the tabular view with which general users are familiar. In contrast, parallel bargrams keep the tabular representation but still support sorting all attributes in parallel rows at the same time [19] and LineUp [20] and SimulSort [21] also visualize the value of a cell with stacked bar charts to help visually sum multiple items. Dimara et al. [22] examined how parallel-coordinate plots, scatterplot matrices, and tabular visualization are effective for analytic tasks involving multi-attribute decision-making. These techniques help users browse through the data by making data accessible rather than making a decision automatically.

Although the majority of research in the field has focused on how to augment human cognition through visualization techniques and tools, recent studies raised an issue about cognitive biases for information visualization [22,23].

### 2.2. Decision-Making Biases and Decoy Effect

H. A. Simon [24] introduced the concept of bounded rationality, which is the basis of behavioral decision research. It was an alternative approach to normative decision theory, assuming that decision makers are fully rational. Simon viewed decision makers as satisficers who seek a satisfactory solution rather than an optimal one. Because human cognitive capacity is limited, decision makers may keep information demands within their cognitive capacity. Due to this, as people make mental shortcuts known as heuristic strategies, in some cases, they lead to cognitive biases that affect the decision-making process, which are systematic errors in judgement.

**Figure 2:**
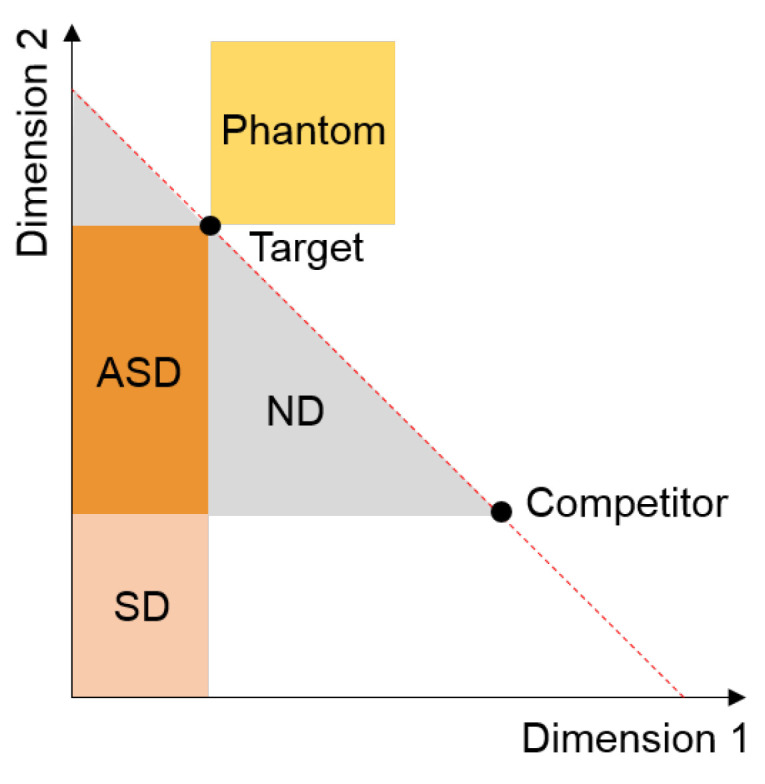
Locations of the two alternatives and decoys.

Among several cognitive biases such as confirmation and anchoring effects [25], the decoy effect is a well documented phenomenon that is used in many choice models and marketing in industry [26,27,28]. The decoy effect appears in a decision-making task that involves a set of options and is characterized by attributes. The decision maker has to make a choice, weighing their preference for each attribute. Given a set of options, the attraction effect refers to an enhancement in the choice probability of an option through the introduction of a similar but inferior alternative called a **decoy**. The three common decoys that are discussed are symmetric dominated, asymmetric dominated, and phantom decoys [29]. Figure 2 graphically depicts these three types of decoys. The two dimensions are attributes that the decision maker should consider and options along the red dotted line are not comparable, which means any option along the line can be optimal. It is a matter of personal preference.

Symmetrically dominated decoys are in the SD region shown in Figure 2. One alternative dominates another when it is clearly superior based on at least on attribute. The symmetric decoy is dominated in both aspects considered by the decision maker. Wedell [30] showed that the effect of the symmetrically dominated decoy is not statistically significant in terms of changing one’s preference structure. For example, considering the context in Figure 1, it would be an option that costs $59.00 and only offers a 6-month subscription. Asymmetrically dominated decoys in the ASD region are shown in Figure 2. An ASD decoy is dominated by one alternative in a set of options but not by the other options. For example, it is the third option, Print & web subscription, in the economist example (Figure 1). The item dominating the decoy is called the **target**, and the other item is the **competitor**. Extensive research has shown that adding an ASD decoy dramatically increases the likelihood that the decision maker will select the target option [29]. Note that the target and competitor are comparable favoring one dimension over the other. These types of decoys are most extensively studied (e.g., [2,30,31]). The choices in the non-dominated region (ND) are not dominated by a certain dimension and are alternatives that can be chosen based on one’s preference.

The phantom decoy was introduced relatively recently [32]. It is a highly attractive option included in the choice set but unavailable at the time of choice. Although the decoy appears to be a better choice because it dominates its target, it is an unattainable choice or is so dominant in an attribute that it becomes unattractive [29]. Although this seems counter-intuitive, it is best explained by the loss aversion principle used in the relative advantage model, which states that losses loom larger than gains [33].

Among the various decoy classes, asymmetrically dominated decoys show the strongest decoy effect and have been studied in many contexts and with a wide range of alternative values, and the reported results are stable and strong [2,30,34,35,36]. In many cases when people refer to decoy effect, it refers to an asymmetric dominance effect. Therefore, our research also focused on the asymmetrically dominated decoy effect.

There are several explanations for why preference reversals occur with a decoy. According to the weight-change model, adding a decoy changes the relative importance of each attribute. Therefore, the preference between target and competitor can be changed [37]. However, several studies found evidence against this model [31,38]. According to the value shift model [39], the changes do not result from the weight change of the attribute, but rather change the decision maker’s evaluation of the attribute values of the target. Both share a common assumption that the decoy effects result from evaluating the value of a target considering both attributes.

The third explanation is the dominance-valuing model. This is based on modern theories of behavioral decision-making that decision makers use heuristic strategies within a cost–benefit framework. A decision maker can adopt strategies to minimize highly cognitive activities or find compelling or simple justifications. With a low selection of decoy options, decision makers easily detect the deteriorated option. Once they have detected the decoy, the decision maker may choose the targeted alternative because it is easier to justify. The dominance-valuing model differs from both the weight-change model and the value-shift model because the value of the option is perceived based on the dominance structure which increases the attractiveness of the target rather than calculating its value from the attributes.

### 2.3. Decision-Making Styles

Decision-making styles, defined as “the learned, habitual response pattern exhibited by an individual when confronted with a decision situation” [40], are another well-known approach to analyzing the individuals’ decision-making behaviors. Decision-making styles are relatively stable and lasting cognitive factors which can be applied to interpret people’s attributes while making choices [41].

Despite their wide use and strong impact in decision-makings, decision-making styles in the field of information visualization have not yet been fully explored. A study [42] has found a significant interaction effect between information visualization techniques and decision-style on task completion time, but little research was undertaken to analyze the main effect of decision-making styles in various information visualization strategies.

There are several approaches to classify the decision-making styles. Herbert Simon [24] proposed the two different types of decision-making: a rational analytical and an intuitive style. According to the study, rational-style people have a tendency to evaluate information with explicit reasoning process, whereas the intuitive-style is more focused on their prior expertise and experience. Another example of decision-making styles is spontaneous, dependent, and avoidant [43].

In this research, we adapted the Scott and Bruce’s (1995) approach of GDMS (General Decision Making styles), which has been widely used in various fields of research [44], including marketing [45] and job decisions [46]. With the 25 items, GDMS distinguishes decision-makers between five categories: (a) Rational: making decisions based on the logical evaluations of alternatives and having a sequential information process; (b) Intuitive: having strong dependence on emotional and gut feelings; (c) Dependent: Reliance on the directions and advice from others; (d) Avoidant: attempts to avoid making decisions since they feel uncomfortable when doing it; and (e) Spontaneous: a tendency to “get through decision-making process as soon as possible”. According to the prior research [47], these styles are also divided into two categories. Rational, intuitive, and spontaneous decision-making styles are distinguished as a “core decision process” which are concerned more with the cognitive way individuals make choices. By contrast, the other two—dependent and avoidant styles—are related to the benefit–risk assessment identified as a “decision-regulatory process”.

### 2.4. Debiasing the Decoy Effect

Although the decoy effect is a well-known cognitive bias, little research has explored how to minimize or decrease the decoy effect. Current research mainly has focused on the underlying for the decoy effect and how to utilize the effect from the marketer’s point of view.

Early work breaking the decoy by Teppan and Felfernig suggested a decoy minimization method to break the decoy effect [48]. Decoy minimization mainly involves two methods: (1) excluding decoys from the alternative set; (2) including counteracting decoys in the set. They suggested including counteracting decoys because the elimination of decoys is hard to accomplish; it can be ambiguous whether an alternative belongs to the set of the decoy or not, especially when the decoy is not dominated [49]. Counteracting decoy methods introduce another decoy of a competitor to neutralize the effect of the existing decoy. Teppan and Felfernig tested the asymmetric dominance effect of decoys through an unsupervised online study [48]. Subjects in the study were asked to evaluate the results of a fight tournament, and each avatar had different mobility, quickness, and punching-power ratings. For two given alternatives as shown in Table 1, their respective asymmetrically dominated decoys were also generated and included as part of the choice set. The experiment featured 11 sets of choices. To neutralize the decoy effect of A- on A, they introduced B—whose mobility was 5, and the power was 2. In the user study, they found that this counteracting decoy reduced the decoy effect.

However, adding another option in the given set of options to decrease the decoy effect is not realistic because it is not a common strategy that a person would normally employ in a real-world setting. Hence, recent work was introduced utilizing visualization with the first approach, excluding decoys from the alternative set [10]. Dimara et al. demonstrated that interactive scatterplots helped to remove locally dominated points. They assumed that interactive scatterplots would help execute elimination by aspect, a common decision-making strategy that helps remove salient inappropriate data to minimize the amount of information to consider. While the study presented some evidence that interactive visualization could mitigate the decoy effect, it considered a relatively large number of options (e.g., 10 options), which far exceeds the common number of three options that most studies used in their experiment. For a three-option setting, scatterplots still replicated the decoy effect [9].

We believe that various visualization techniques should be investigated to see whether they can help to alleviate the decoy effect with more realistic datasets.

## 3. Methods

Two experiments were conducted. The first crowdsourcing experiment was to run a quantitative analysis on the decoy effect and examine if visual interfaces could mitigate cognitive biases. The second qualitative study was conducted with a semi-structured interview to get a deeper understanding of the users’ decision-making strategies. The same data sets and visualizations were used for both studies.

### 3.1. Design Rational

#### 3.1.1. Data Sets

To create data sets, we considered both different scenarios and proper attribute values for each context. First, several scenarios have been used in decision-making studies. One of criteria we used for the scenario is that it needs to have a price range as an attribute. Because price is an attribute for which most people have a similar preference (e.g., the cheaper, the better), we assumed that it would minimize individual differences in preference. We also wanted to include different contexts and attribute values in the experiment to consider diverse decision-making situations. The resulting four scenarios capture decision-making contexts that commonly occur in daily life; subscribing to a magazine, subscribing to a video streaming service, purchasing coffee gift cards, and selecting a data plan for a cell phone. Each scenario consisted of two attribute dimensions for making a decision (e.g., price and number of cups of coffee) (see Table 2).

Second, we needed numerical values for each attribute. To replicate the scenario shown in Figure 1, as it is the most well known scenario for decoy effect experiments, we needed to change the subscription type (i.e., web and paper) to numerical values, done in several previous studies (e.g., [36,50]). Therefore, in addition to the price attribute, we selected the number of issues per year. The range for this attribute was determined by the unit price per issue. To control the level of difficulty, the unit price per issue was set to be close. For example, for the economist magazine, the unit prices for the competitor, target, and decoy were $2.80, $2.40, and $2.97, respectively (see Table 2). However, when spending more money, the unit price was lower per item, which indicates a greater benefit from spending more. The target is Option B, with a high price and more issues. The competitor is Option A, with a lower price and fewer issues. Option C is the decoy for Option B and has the same price but fewer issues. We positioned the decoy at the end for two scenarios and in the middle for two scenarios to minimize the order effect. For other attribute values, we tried to incorporate the values from the real world so it would be realistic to the participants.

We also added a filtering question to maintain quality of the data as we conducted an crowdsourcing experiment. The options are designed to be obvious so that, if they answered the questions wrong, we could assume that the participant was not paying proper attention to the task. The task was to make a choice for gym membership that had two options such as $290 for 24 months and $365 for 12 months. It would make sense to select the first option as it is cheaper, but one gets a longer duration.

#### 3.1.2. Stimuli

**Figure 3:**
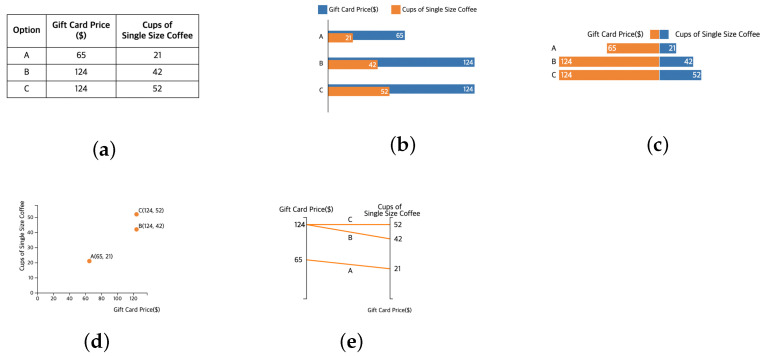
Examples of experimental stimuli. Four visualizations and a table for a baseline: (**a**) Table, (**b**) One-Sided Bar Chart, (**c**) Two-Sided Bar Chart, (**d**) Scatterplots, and (**e**) Parallel-Coordinate Plot.

To investigate whether the visual representation styles may influence the attraction effect, we considered five types of representations: table, one-sided bar chart, two-sided bar chart, scatterplots, and parallel-coordinate plot (See Figure 3). For the stimuli selection, we considered familiarity of the visualization based on education and exposure through media [51] and effectiveness shown in the visualization community. The table was selected for the baseline because most previous work has represented the data in a tabular form with text; we also wanted to determine if we could replicate decoy effects with our data sets.

Scatterplots were used in a previous study to debias decoy effects [10]. The researchers selected 2D scatterplots because they are suitable for presenting data with two dimensions, and the most common is the attraction effect literature (e.g., [2,5]), which means that the representation could help consumers find the decoy easily. Although scatterplots have the advantage of visualizing quantitative values, even for large amounts of data, they are not a common representation seen in everyday life. They are often used for statistical purposes. Therefore, we also selected more common representations.

Our criteria for selecting the visualizations to represent the data were that it had to (1) handle different attributes, and (2) make each alternative comparable. The first common choice was a bar chart. Bar charts are familiar to the general public because they are taught in the K12 curriculum and are well known for supporting comparison tasks [51,52,53]. Because there are two attributes for an alternative, we needed to layer two bars on the same side. To help detect each attribute visually, we color-coded each attribute. However, layering two attributes on the same side for an alternative has limitations because each attribute is measured by a different unit. It could be misleading and, if the numeric values differ significantly, the attribute with smaller values may not appear properly. To overcome this issue, we made a variation of the one-sided bar chart, creating a two-sided bar chart in which the bars were split into two directions. This approach placed the attributes opposite each other from a central axis. This contrast allowed the attributes to be compared easily while still showing the relative size of each alternative.

Last, we chose parallel-coordinate plots. Although it is not common to use parallel-coordinate plots for data sets with only two attributes and three alternatives, they suit the task of comparing attributes for alternatives. Additionally, because the decoy is dominated by the target especially considering one of the attributes, the delta (angle) of the lines connecting the attributes will be different. We tested whether this has influence while comparing alternatives.

To examine whether visualization types can mitigate the decoy effect, we conducted an experiment through a crowdsourcing platform Amazon Mechanical Turk (MTurk). The crowdsourcing approach has several advantages over conventional, controlled laboratory studies [11], including recruiting a large number of participants with diverse backgrounds, and it has been used in several information visualization experiments [14,54].

### 3.2. Crowdsourcing Experiment

#### 3.2.1. Experimental Design

**Figure 4:**
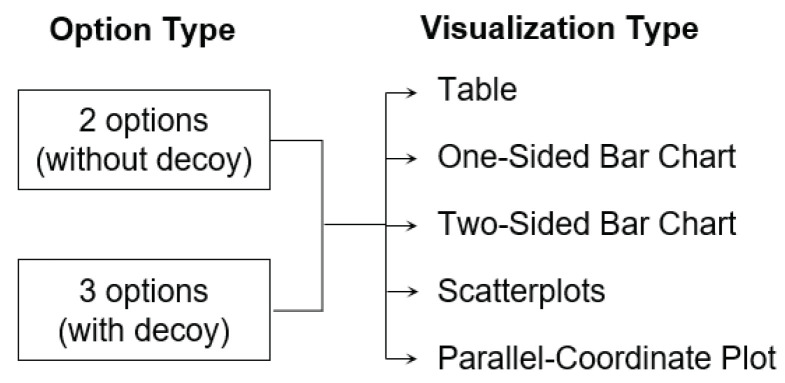
Between subject design with two factors, option type and visualization type.

We conducted a between subject design with two factors; an option-type with two levels with and without decoy; and a visualization-type with five levels: table, one-sided bar, two-sided bar, scatterplots, and parallel coordinates plot (see Figure 4). To evaluate the attraction effect, participants from the control group were asked to choose the most attractive option between the two alternatives (i.e., target and competitor). For the experimental group, three-alternatives were presented (i.e., target, competitor, and decoy).

#### 3.2.2. Participants

A total of 576 participants were originally recruited through MTurk. The workers’ requirements to participate in our task on MTurk was an HIT approval rate greater than 95%, located from the United States, and more than 500 approved HITs. We found that 107 were identified participants as insecure, and they did not pass our filtering question, which was asking to make an obvious decision (see Section 3.1.1 for details). One participant of the scatter-plot condition was removed, since the participant gave less than two points at all given four tasks, which means that the person did not understand how to read the visualization. The remaining, legitimate participants (n = 468) were divided among the condition; each condition had at least 43 participants and the condition with the most participants had 52. Among the 469 participants, 221 were female and 248 were male with a self-reported age range of 20 to 77; and an average age of 37.8. None of them participated in more than one condition. The education levels of the participants were as follows: bachelor’s degree in college, 42.0%; some college but no degree, 20.0%; high school graduate, 12.3%; and associate’s degree; 11.5%. The baseline payment for participation was $0.30. An additional bonus reward was $1.00.

#### 3.2.3. Tasks

**Figure 5:**
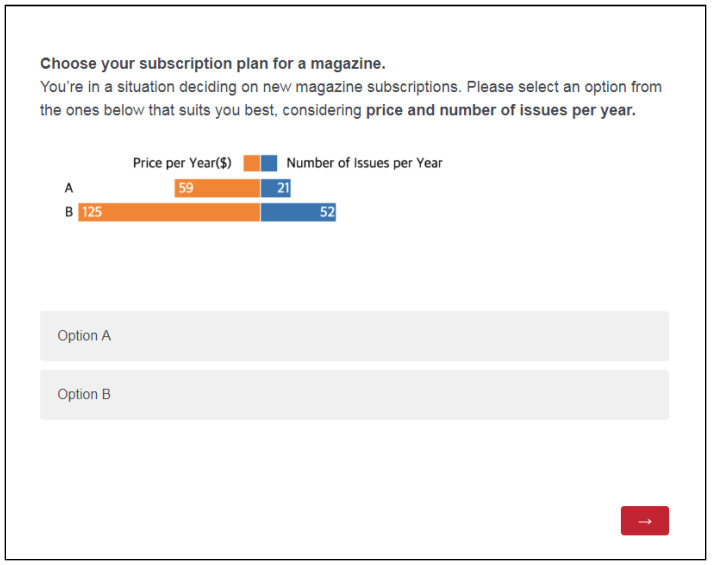
Capture of experimental website with a two-sided bar chart with two options without decoy.

The task was to select the best choice of the given options. After selecting one, the participants had to rate their preference on a scale of 0 to 10. The experimental website is shown in Figure 5.

#### 3.2.4. Measures

Four types of quantitative data were collected to answer research questions. Since the attraction effect was evaluated based on the proportions of choosing alternatives [2], we first collected the choice proportions of each alternatives. Next, decision competence was measured with a seven-point Likert-type questionnaire to confirm that the participants deliberately took part in the experiment. In addition, a questionnaire was included asking the reading ability of each visualization type, which was scored on the seven-point Likert scale. Furthermore, the GDMS instrument [44], comprised of 25 items, was used to identify the decision-making styles of participants. The GDMS scale, a reliable and valid scale for assessing decision-making [55], consisted of the seven-point Likert scale questions with five items allocated for each style: (1) rational (e.g., “ I double-check my information sources to be sure I have the right facts before making a decision”); (2) intuitive (e.g., “When making a decision, I rely upon my instincts”; (3) spontaneous (e.g., “I make quick decisions”); (4) dependent (e.g., “I use the advice of other people in making important decisions”); and (5) avoidant (e.g., “I generally make important decisions at the last minute”). Given that the experiment consisted of the tasks where participants supposed to make decisions by themselves, three of the ‘core decision-making process’ styles (rational, intuitive and spontaneous) were analyzed in this research.

#### 3.2.5. Procedure

The experiment began with a tutorial briefing the participants about the study details, including the compensation policy. Then, they faced four scenarios with choice-making tasks, selecting the most attractive alternative among the options presented. In terms of order, the scenarios were randomly shown to each participant. After each decision-making task, they filled out two survey questionnaires evaluating their confidence level of the decision and clarifying their reading competence of the assigned visualization chart. Furthermore, a screening question was given to make sure that participants were paying attention and included between the decision-making tasks. After finishing all decision-making tasks, the participants filled out a demographic survey and 25 items of the General Decision-making Style (GDMS) test. Finally, an open question survey was conducted asking about the participants’ decision-making strategy to gain a deeper understanding of participants’ decision-making strategies and their difficulties during the experiment.

#### 3.2.6. Hypotheses

Given the aforementioned results from the literature review, the research hypotheses are as follows:

**Hypothesis** **1.** *A larger proportion of participants will choose the target when the decoy is present.*

**Hypothesis** **2.** *The decoy will have differing influences depending on the format of visual presentations.*

**Hypothesis** **3.** *The attraction effect will be influenced by decision-making characteristics of people.*

### 3.3. Qualitative Experiment

To gain a deeper understanding of decision-making strategies and reasons for the attraction effect, we conducted a semi-structured interview with the same survey we used in the experiment. We also analyzed the open-ended question we asked to explain the strategy participants used in the crowdsourcing experiment.

#### 3.3.1. Participants

We recruited eight participants for the interview (mean age = 20.25, σ=0.88, and five were female). All were undergraduate students recruited from two classes at a University in South Korea and they took part in the interview voluntarily.

#### 3.3.2. Procedure

The interview began with a short description of the research purpose and procedure. Then, participants were asked to conduct the decision tasks via the online survey website, to be consistent with the prior experiment. After that, the semi-structured interview was conducted. During the interview, we asked participants a few questions about their decision-making strategies and impressions regarding the decision tasks to check their familiarity with each task. We also asked participants how they would describe themselves among three core decision-making styles, to investigate the influence on their decisions.

## 4. Results

### 4.1. Crowdsourcing Experiment

As previous studies suggested [56], a chi-square test was used to evaluate the attraction effect. A chi-square test informs whether or not there is a statistically significant difference between different segments, in our case the presence of the decoy option, especially when the data are presented in a cross tabulation form. For the first step, we calculated descriptive statistics to analyze the characteristics of collected choices. Among 1876 choices, the decoy selection rate was reported as 4.5% on average, which is relatively low compared to previous studies [3]. Consistent with previous studies [9], choices selecting the decoy were removed from the analysis.

#### 4.1.1. Presence of Decoy

Overall, decoy appeared to have a strong effect on the decision-making process. There was a significant difference between option-types ($\chi^{2}=24.780$, p<0.001), which shows the strong attraction effect. More specifically, the choice changed, with 64.8% choosing the target in the decoy condition and 53.8% choosing it the without the decoy. These results indicate that people are highly affected by the presence of a decoy when making decisions.

However, mixed results were found across the decision tasks. As shown in Table 3, the attraction effect was appeared in three tasks (video streaming, magazine subscription, and coffee gift card), while there was no statistical difference in the phone data plan task. Among these three tasks with positive results, a strong attraction effect was shown in the coffee task. For example, the proportion of participants selecting the target in the coffee task was more than 55%, an increase of 8.4% compared to the without-decoy condition (p=0.001). Meanwhile, even though there was no statistical change in choices pattern in the coffee and data plan task, the target selection rates lightly increased from 57.3% (without-decoy) to 58.5% (with decoy). Hence, H1 was partially supported.

#### 4.1.2. Visualization Type

**Figure 6:**
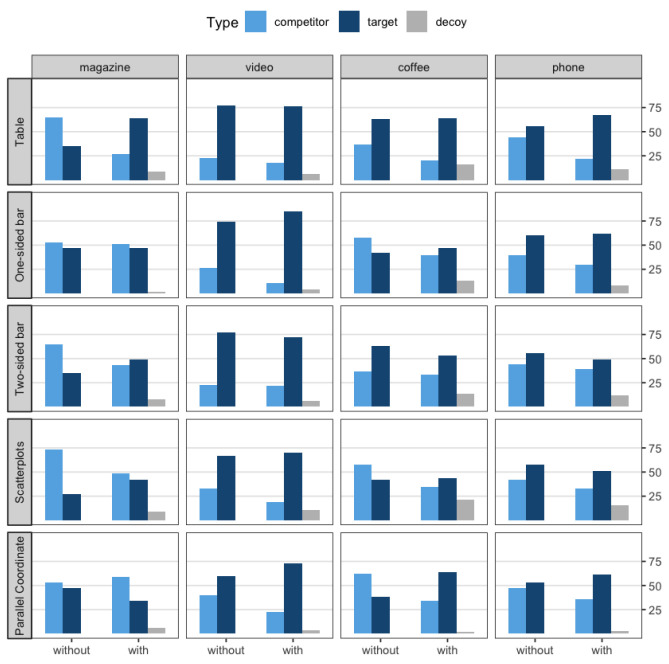
Percentage of selection of each option across all scenarios and visualizations.

Mixed results were found across the five visualization types. A significant difference was found in three visualization conditions (i.e., table, scatterplots, and parallel-coordinate plots), but no statistical differences emerged in the other two conditions (i.e., one-sided and two-sided bar), meaning that there was no decoy effect. A strong effect was reported especially in the table condition ($\chi^{2}=17.611$, p=0.000) (see Table 4). In addition, the target had a greater share in the decoy × scatterplots and with-decoy × parallel-coordinates plot condition. Similar to these results, although there was no statistical significance, participants in the one-sided bar condition had a tendency to select the target option rather than the competitor when the decoy was present (p=0.077). By contrast, there was no significant change in the choices pattern of the two-sided bar condition, regardless of the presence of a third option (decoy). The overall comparison of the selection of the three options is shown in Figure 6.

#### 4.1.3. Decision-Making Style

We performed a logistic regression to evaluate the influence of the individuals’ decision-making style (e.g., rational, intuitive, and spontaneous styles) on the attraction effect. As a result, the Nagelkerke $R^{2}$ was reported as 0.013, indicating that the logistic model can not appropriately explain the effects of dependent variables (decision-making styles) on the independent variables (the attraction effect). Thus, H3 was not supported.

#### 4.1.4. Confidence Level of Choice

The mean decision-confidence level of participants in their choices was 5.9 (σ=1.10) on a 7-point Likert scale, and there was no big difference in these five visualization-types (Min = 5.82, Max = 6.01) and in four scenarios (Min = 5.76, Max = 5.97). This indicates that overall participants had confidence in their decisions.

#### 4.1.5. Level of Visualization Comprehension

Lastly, we analyzed the self reported mean reading-comprehension level on each visualization-type. The mean level was reported as 6.15 (Min = 6.00, Max = 6.34, among five visualization-types), indicating that most participants were able to read their assigned visual representation. There was no statistical difference among the mean rating for the visualizations: table (μ=6.26, σ=0.93), one-sided bar (μ=6.34, σ=0.92), two-sided bar (μ=6.11, σ=0.98), scatterplots (μ=6.00, σ=1.23), and parallel-coordinate plots (μ=6.02, σ=1.14).

### 4.2. Qualitative Experiment

Participants mainly took two factors into accounts when making decisions: unit price and their preference for a certain attribute. During the interview, three participants reported that they made decisions regarding their preference on a certain attribute. For example, one of the participants responded that she selected the option with the shortest time period of magazine subscriptions, factoring in her usual buying habits. Consistent with qualitative findings, some participants reported that they first considered the amount of data they usually used to make decisions on phone data plans; therefore, if they needed more than 5 GB, they would select the option with the highest amount without considering the price.

*“When choosing a service or a product, I usually like to experience it briefly first. Therefore, in this case, I chose the option with the shortest time period over the price”*—p5

In contrast, three out of eight participants from the interview indicated that the unit price was the most important factor when making decisions. They would calculate the unit price first and selected an option that would maximize their cost effectiveness.

*“Usually I think of it as units. I compared the price of each unit first and choose the most beneficial one”*—p2

**Figure 7:**
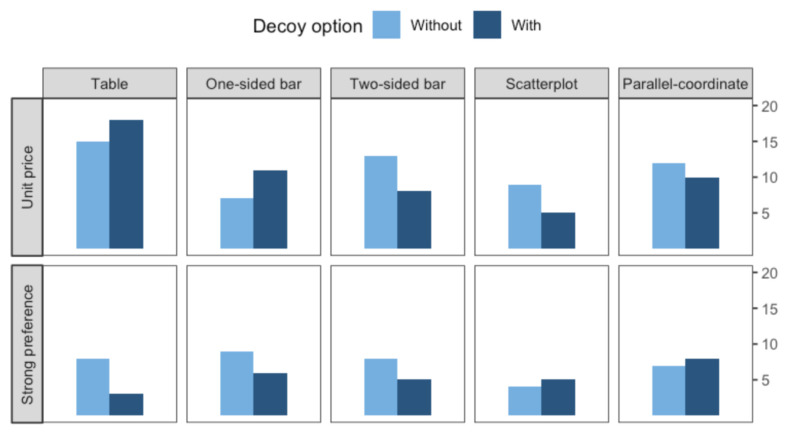
Frequency of each strategy mentioned by the participants in the interview.

A similar tendency was found in the responses of the open-question survey which was included in the crowdsourcing experiment. Figure 7 shows two main decision-making strategies people mentioned a lot during the interview: unit price and individual preferences. We also reported how frequently they mentioned specific strategies. As shown in the figure, calculating unit price appeared to be the most common strategy that people took while making decisions. Some of the participants quickly glanced at the price, while others actually went through a price comparison. In addition, several participants mentioned that they used the ‘double-count’ strategy where they doubled the price of the cheaper option and compared it with other alternatives.

*“I was first considering the price value of the package, such as dollar per unit or session”*

*“I tried to get the most for my money. I tried to see if it was cheaper for [option] 2 if [option] 1 were doubled.”*

Aside from these numerical strategies, there were participants who explicitly used visualization as one of their decision-making strategies. For example, a participant reported that they used a bar-chart as the ‘virtual ruler’ to compare the options with the naked eyes.

*“I usually divided blue portion by the orange portion and went with which one was more.”*

*“I determined the monthly price based on PPY [price per year] and what was provided. The charts provided a pretty accurate representation of those numbers/values.”*

On the other hand, there were some negative comments on scatterplots. Some participants responded that scatterplots were not as helpful in making decisions.

*“I pretty much ignored the diagram as it didn’t make too much sense.”*

*“I mostly wanted to get the best amount of service for my dollar. Having to refer to a chart made it a bit more difficult to decipher how much I was spending.”*

## 5. Discussion

### 5.1. Effect of Visualization Types on Decoy Effect

Initially, we hypothesized that visual interfaces may be used to decrease the decoy effect, and our study proved that specific types of visualizations help people avoid the decoy. Among the five types of visualizations, one-sided bar charts and two-sided bar charts turned out to be effective in preventing the decoy effect; we suspect this is because these two visualizations supported their decision-making strategy. From the qualitative analysis, we found the most common strategy people employed to calculate unit price and that they were able to measure the unit price more easily using one-sided and two-sided bar charts, with the help of a visual indicator (i.e., bar size).

Interestingly, we initially hypothesized that parallel-coordinate plots would be most effective in minimizing the decoy effect because the slope could be intuitive for calculating the unit price or comparing relative values. This was not statistically proven, possibly due to unfamiliarity with the visualization type. People are not typically familiar with parallel-coordinate plots and thus are not good at interpreting them, which might have negatively affected their decision-making process [57].

Scatterplots did not seem to help avoid the decoy effect in our experimental setting. They did not support people’s decision-making strategy, and a few participants expressed a negative reaction to the visualization itself because they felt it did not make sense, and it made it hard to decipher the values.

To gain a better explanation on understanding on the underlying process of why certain visualizations help the decision-making process, we examined graph comprehension models [58,59,60,61]. According to the models, the performance for a task while using a visualization is attributed to the match between the task and the visualization. If the necessary information to accomplish the task can be extracted directly from the visualization, only perceptual processing is required (e.g., retrieving a value from a bar chart). However, if the necessary information cannot be extracted directly, spatial processing may be required.

In general, spatial cognition tasks can be solved by a non-spatial strategy, usually mathematically, but people prefer spatial strategies if the visualization is properly given [60]. In our study, the majority of the participants mentioned that the decision-making tasks required mathematical calculation. Since the mathematical calculation is known to be cognitively challenging, people may have fallen into a decision-making bias, applying it as a strategy within the cost–benefit framework (see Section 2.2), and justifying their decision by saying that the target option was at least better than the decoy. However, with proper visualizations, namely one-sided and two-sided bar charts, the task might have been accomplished with spatial processing. That is, with the bar charts, calculating the proportion and comparing values might have offloaded the mental load, helping the participants make a better decision.

### 5.2. Effect of Scenarios on the Decoy Effect

It turned out that different task types, or scenarios, do affect the decoy effect. While relatively ”light" scenarios such as video streaming, magazine subscriptions, and coffee gift cards showed the decoy effect, the phone data plan task did not exhibit the same pattern. In other words, people were not affected by the decoy in choosing the data plan. One possible explanation is that a relatively large number of people have a strong preference for data size, which might have affected their decision-making process. That is, those people would choose the option with the largest data size no matter how much it costs, which weakens the influence of the decoy.

### 5.3. Effect of Decision-Making Style on the Decoy Effect

Different decision-making styles may affect an individual’s degree of cognitive bias. While we examined the relationship between decision-making styles (e.g., rational, intuitive, and spontaneous) and the decoy effect, no statistical significance was found. In other words, even when a person has a rational decision-making style, he or she is still prone to cognitive bias such as the decoy effect in decision-making.

It was also not explored whether certain types of decision-making styles may be better supported by visual interfaces, and it would be interesting to further examine how individual differences in decision-making influence cognitive bias and where in the process visualizations might help.

### 5.4. Limitations

Our study has several limitations. First, for experiments to observe a decoy effect, both between-subject and within-subject experiments had been conducted in previous research. To be more conservative in capturing the effectiveness of the visual representations, we conducted a between-subject study with the advantage of avoiding carryover effects. The participants only experienced one type of visual representation and one condition for the presence of a decoy. However, as decoy effect is about capturing the change in preference to attributes, if we want to directly measure whether a person changes his or her choice in the presence of a decoy, a within-subject study needs to be employed. If this was the case, we could have had a better understanding of whether individuals’ decision-making style had an impact on the decoy effect.

While we tried to incorporate different visualization types in our experiment, testing with more diverse visual interfaces would yield other meaningful insights. We also only focused on the case in which we had three options. If the number of options increases, the effectiveness of visualization could change.

As mentioned previously, it turned out that decision-making scenarios affect the results, which makes it hard to generalize the findings to all decision-making contexts. However, this is an unavoidable problem in decision-making studies because so many different cases exist in our everyday decision-making. Therefore, for further research, we could focus on a certain market segmentation for a limited context to avoid this problem. Additionally, even though we tried to create scenarios that reflected common decision-making situations in everyday life, some participants found them irrelevant. For example, some participants mentioned that they do not read magazines, so they will select the cheapest one. This might have decreased the ecological validity of the study.

## 6. Conclusions

In this study, we sought to examine whether the decoy effect, a well-known cognitive bias in decision-making, could be mitigated with the help of visual interfaces. After conducting a crowdsourcing experiment using Mturk, we analyzed quantitative data from 469 participants, as well as qualitative data from open-ended questions and follow-up interviews. The results showed that, while different decision-making scenarios may affect the decoy effect, certain types of visualizations such as one-sided and two-sided bar charts help alleviate the decoy effect in decision-making. We believe that this research could be the first step towards uncovering the role of visualization in decreasing cognitive biases, eventually helping people make more informed decisions.

Not only for marketing, decoy effect and visualization can be used to help users make better decisions in everyday life. Consumers are exposed to several situations to compare attributes and make a choice such as for selecting a meal plan or a work-out plan. These representations could help to make a better choice. The use of visualizations is increasing on websites and mobile apps and are known to lower the cognitive load even for processing more information if properly presented [15]. Therefore, we believe that further research on cognitive biases and proper visualizations could help with the decision-making process for various situations.

## Figures and Tables

**Table 1:** Example of Primary Alternatives and a Decoy [48] (p. 24).

	Mobility	Power
Character A	3	8
Character A-	1	6
Character B	7	4
Character B-	5	2

**Table 2:** Data set for each scenario.

Scenario	Options	Attributes		Type
Economist Magazine		Price per Year ($)	Number of Issues	
	A	59	21	Competitor
	B	125	52	Target
	C	125	42	Decoy
Video Streaming Service		Price ($)	Duration (months)	
	A	47.99	6	Competitor
	B	78.99	12	Target
	C	78.99	10	Decoy
Gift Card for Coffee		Price ($)	Number of Cups	
	A	65	21	Competitor
	B	124	42	Decoy
	C	124	52	Target
Phone Data Plan		Price per Month ($)	Data Plan (GB)	
	A	28.99	5	Competitor
	B	48.99	8	Decoy
	C	48.99	10	Target

**Table 3:** Summary of choice probabilities for the decoy placement in each scenario.

Scenario	Decoy Option	Competitor	Target	Decoy	$\chi^{2}$	*p*-Value
Economist Magazine	Without	62.3%	37.7%		8.35	0.004
	With	45.4%	47.2%	7.4%		
Video Streaming Service	Without	28.9%	71.1%		5.34	0.021
	With	18.3%	75.1%	6.6%		
Gift Card for Coffee	Without	53.1%	46.9%		11.303	0.001
	With	32.6%	55.3%	12.1%		
Phone Data Plan	Without	42.7%	57.3%		2.471	0.116
	With	32.3%	58.5%	9.2%		

**Table 4:** Summary of choice probabilities for decoy placement for each visualization.

Visualization	Decoy Option	Competitor	Target	Decoy	$\chi^{2}$	*p*-Value
Table	Without	45.3%	54.7%		17.611	0.000
	With	21.7%	67.8%	10.5%		
One-sided Bar	Without	44.3%	55.7%		3.134	0.077
	With	32.5%	60.1%	7.4%		
Two-sided Bar	Without	42.3%	57.7%		0.738	0.390
	With	34.3%	55.9%	9.8%		
Scatterplots	Without	51.6%	48.4%		5.392	0.020
	With	33.7%	51.7%	14.6%		
Parallel-coordinate Plots	Without	50.5%	49.5%		4.256	0.039
	With	38.0%	58.0%	4%		

## Data Availability

The data presented in this study are available on request from the corresponding author.
